# Characteristics of longitudinal changes in quality of life and associated factors in patients post cardiac and thoracic aortic surgery: insights from a prospective cohort study

**DOI:** 10.1186/s41687-024-00787-9

**Published:** 2024-09-26

**Authors:** Masaaki Sato, Hitoshi Mutai, Shuhei Yamamoto, Daichi Tsukakoshi, Keisuke Furuhashi, Hajime Ichimura, Yuko Wada, Tatsuichiro Seto, Hiroshi Horiuchi

**Affiliations:** 1 Division of Occupational Therapy, School of Health Sciences, Shinshu University, Matsumoto, Japan; 2 Department of Rehabilitation, Shinshu University Hospital, Matsumoto, Japan; 3 Division of Cardiovascular Surgery, Department of Surgery, Shinshu University School of Medicine, Matsumoto, Japan

**Keywords:** Quality of life, Cognitive impairment, Activities of daily living, Cardiovascular surgery, Cardiac rehabilitation

## Abstract

**Background:**

Although quality of life (QOL) is an outcome of postoperative cardiac rehabilitation (CR), its course and related factors from postoperative hospitalization to the post-discharge period have not been adequately investigated. Additionally, the EuroQol-5Dimension-5Level (EQ-5D-5L) index score has not been characterized over the same period. We aimed to characterize QOL changes assessed by the EQ-5D-5L, over the period from hospitalization to 1 year post-discharge, in patients post-cardiac and thoracic aortic surgery, and investigate the factors associated with these temporal changes.

**Methodology:**

This prospective, single-center study included 117 patients who underwent open cardiovascular surgery (median age, 72 years; men, 69%). Patients were assessed for QOL status when transferred to the general ward; at discharge; and at 6 and 12 months after discharge, using the EQ-5D-5L index score and a generalized linear mixed model with random intercepts. Patients were classified into two groups based on score changes post-discharge. Logistic regression analysis evaluated factors associated with QOL decrease post-discharge.

**Results:**

The EQ-5D-5L index score significantly increased over time, except between 6 and 12 months post-discharge; “Common activities” was the most common dimension showing score improvement. In 25 patients (21%), the EQ-5D-5L index scores were lower after discharge compared to their scores at discharge. In the logistic regression analysis, Barthel Index pre-admission, preoperative hemoglobin level, and Mini-Mental State Examination-Japanese scores pre-discharge were significantly associated with QOL decline after adjusting for the European System for Cardiac Operative Risk Evaluation II score.

**Conclusions:**

Most patients post-cardiac or thoracic aortic surgery experienced improved QOL from postoperative hospital stay to 1 year post-discharge. However, in patients with pre-operative basic activities of daily living, hemoglobin and post-operative cognitive decline may require ongoing comprehensive CR because of reduced QOL. Given the potential selection bias introduced by the relatively small sample size in this study, future research involving larger populations is necessary.

**Supplementary Information:**

The online version contains supplementary material available at 10.1186/s41687-024-00787-9.

## Background

The number of cardiac and thoracic aortic surgeries performed in Japan has increased remarkably over the last 30 years [1], particularly among the elderly owing to the rapid aging of the population [2, 3]. This has underscored the importance of cardiac rehabilitation (CR), which aims to improve exercise tolerance, prognosis, quality of life (QOL) [4], and basic and instrumental activities of daily living (ADL) [5, 6]. While CR in Japan is provided by a multidisciplinary team in the early postoperative period based on established guidelines [7], interventions typically cease on hospital discharge. The rate of outpatient CR among patients undergoing postoperative cardiac surgery is lower in Japan than that in other countries [8]. Therefore, some major outcomes of CR, such as QOL and ADL, after discharge in patients undergoing cardiac and thoracic aortic surgery remain largely unexplored.

QOL, an outcome indicator as crucial as prognosis, has been the subject of few studies [9–11] examining long-term QOL changes after cardiac and thoracic aortic surgery. In addition, there are no studies that have revealed changes in QOL over time in patients after cardiac and thoracic aortic surgery using the EuroQol-5Dimension-5Level (EQ-5D-5L)—a common measure used to calculate the utility index [12]. As CR aims to improve the QOL and long-term prognosis of patients with cardiovascular disease, it is important to understand the actual changes in QOL over time in the mid- to long-term post-discharge. In addition, several factors have been associated with QOL of patients after cardiac and thoracic aortic surgery, including age [9, 11], sex [13, 14], diabetes mellitus [11], preoperative cardiac function [13, 15], all of which are included in the European System for Cardiac Operative Risk Evaluation (EuroSCORE) II score [9, 16]. Postoperative factors include mechanical ventilation (MV) for > 48 h [17] and renal replacement therapy (RRT) for acute kidney injury (AKI) [17]. Patients’ mental status, such as depressive symptoms, has also been reported to be a factor related to QOL [18, 19]. However, these studies typically focus on a single postoperative point in time, and factors related to changes in QOL (e.g., improvement, maintenance, and decline) over time are unknown. In addition, the relationships between physical and cognitive functions, ADL assessed during rehabilitation, and postoperative QOL have not yet been clarified. Identifying the characteristics of patients’ postoperative QOL over time and the factors associated with these changes will provide important insights into comprehensive postoperative CR.

Therefore, the purpose of this study was to characterize changes in QOL over time, beginning from hospitalization to 1 year after discharge, in patients undergoing cardiac and thoracic aortic surgery, and to investigate factors associated with these temporal changes.

## Methods

### Design and setting

This prospective cohort study was conducted at the Shinshu University Hospital in accordance with the Strengthening the Reporting of Observational Studies in Epidemiology (STROBE) guidelines [20]. This hospital, which specializes in acute-phase care, provided postoperative CR during hospitalization to patients who underwent cardiac and thoracic aortic surgery. A multidisciplinary team, comprising a doctor, nurse, physiotherapist, occupational therapist, speech-language therapist, and clinical engineering technologist, initiated postoperative CR immediately after admission to the intensive care unit (ICU), with a focus on risk management. The CR program consisted of the following interventions: early mobilization, pulmonary rehabilitation, delirium prevention, aerobic exercises, resistance training, ADL training, and patient education, all in line with the Japanese Circulation Society’s guidelines for the rehabilitation of inpatients with cardiovascular disease [7].

### Participants

**Fig. 1:**
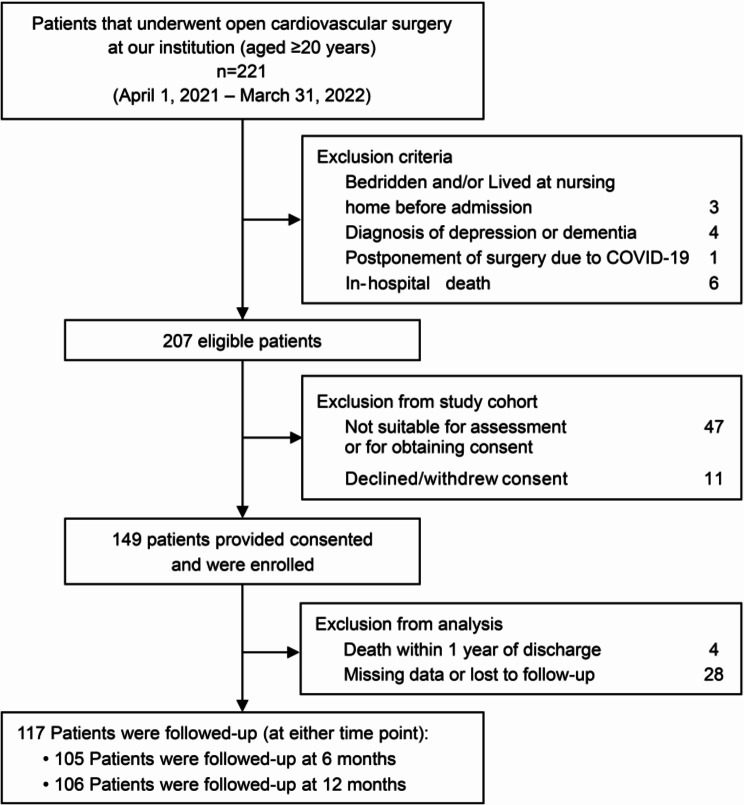
Flow diagram of the study

All patients aged ≥ 20 years who underwent cardiac and thoracic aortic surgery via an open procedure at Shinshu University Hospital between April 1, 2021, and March 31, 2022, were included in this study. During this study period, 221 consecutive patients underwent open cardiac and thoracic aortic surgery. After applying the exclusion criteria and excluding patients who did not provide consent to participate in the study, those with missing data, or those who dropped out during the follow-up period, 117 patients were included in the analysis (Fig. 1). Patients underwent one of the following open procedures: coronary artery bypass, valvular surgery, surgical repair of the thoracic aorta, combined surgery, or other procedures (four patients underwent benign cardiac tumor resection and one patient underwent pulmonary endarterectomy).

### Characteristics of the participants

Patient characteristics during hospitalization, including preoperative status, surgical parameters, and postoperative course, were collected from the electronic medical records and questionnaires. Preoperative variables included age, sex, body mass index (BMI), prior cardiovascular surgery (yes/no), premorbid Barthel Index (BI), comorbidities, estimated glomerular filtration rate (eGFR), blood hemoglobin (Hb) concentration, serum albumin, and EuroSCORE II. Comorbidities were assessed using the Charlson Comorbidity Index (CCI). Preoperative data were obtained within 1 week before surgery. Surgical parameters included the type of operation (elective/urgent/emergent), type of procedure, operative time, cardiopulmonary bypass (CPB) time, cross-clamp time, amount of bleeding, amount of red blood cells (RBCs) transfused, and Acute Physiologic and Chronic Health Evaluation II (APACHE II) score at ICU admission. The postoperative course variables included the duration from surgery to extubation, implementation of RRT for AKI (yes/no), duration from surgery to initial mobilization, incidence of postoperative delirium in the ICU (yes/no), length of ICU stay, length of hospital stay, short physical performance battery (SPPB) before discharge, Mini-Mental State Examination-Japanese (MMSE-J) before discharge, BI at discharge, and discharge destination (home discharge or transfer to a rehabilitation hospital). Initial mobilization was defined as level 2 (passive lift or slide transfer to the chair, with no standing or sitting on the edge of the bed) or level 3 (sitting over the edge of the bed) on the ICU mobility scale [21]. Postoperative delirium was assessed using the Intensive Care Delirium Screening Checklist (ICDSC) [22], and a score of ≥ 4 was considered as “delirium” if it occurred more than once during the ICU stay [22].

### QOL assessment

The EuroQol-5Dimension-5Level (EQ-5D-5L) index was used to measure QOL. The EQ-5D-5L consists of five dimensions related to “mobility,” “self-care,” “common activities,” “pain/discomfort,” and “anxiety/depression,” with patients responding to each item on a scale of 1 to 5 (1 = no problems, 2 = slight problems, 3 = moderate problems, 4 = severe problems, and 5 = extreme problems) [23]. The EQ-5D-5L index score ranges from 0.025 to 1.00 (full health status) and can be calculated to reflect Japanese values [12]. The EQ-5D has also been reported to be reliable and valid as a QOL assessment index in patients with cardiovascular disease [24]. We assessed QOL using the EQ-5D-5L at the following four time points: at the time of transfer to the general ward, at discharge from our hospital, at 6 months post-discharge, and at 12 months post-discharge.

### Follow-up after discharge from our hospital

After postoperative care and medical treatment, the patients in a stable general condition were discharged from our hospital. Patients whose ADL levels did not recover to pre-admission levels due to postoperative complications and/or disuse syndrome were considered for transfer to another hospital to continue rehabilitation and social support management upon completion of postoperative care and medical treatment. We sent mailed questionnaires to patients discharged directly from our hospital as well as those transferred to another hospital, administering them at 6 and 12 months post-discharge. Questionnaires were mailed to the patients for completion and were returned by either the patients themselves or their representatives if they were unable to reply themselves. The follow-up surveys included survival status, ADL performance, and disease self-management measures, in addition to post-discharge QOL. The performance of basic ADL was assessed using the BI, which has also been reported to be reliable and valid for self-reporting [25]. Instrumental ADL (I-ADL) was assessed using the Frenchay Activity Index (FAI). The FAI contains 15 items categorized as follows: domestic (preparing meals, washing up, washing clothes, and light or heavy housework), outdoor (local shopping, walking outdoors, driving/train travel, gardening, and house/car maintenance), and leisure/work (social outings, pursuing hobbies, outings/car rides, reading books, and gainful work). Each item is scored on a scale of 0 to 3 for a total of 45 points, with higher values indicating more frequent I-ADL performance. The Japanese version of the European Heart Failure Self-care Behavior Scale (EHFScBS) [26] was used to assess disease self-management measures. The EHFScBS is a 12-item self-administered questionnaire that covers items concerning self-care behavior in patients with heart failure. The response to each item was scored on a 5-point Likert scale from 1 (“I completely agree”) to 5 (“I do not agree at all”). The total score, which ranged from 12 to 60 points, was calculated by calculating the sum of the ratings for each item. Higher scores indicated poorer self-care behavior [26].

### Statistical analysis

Normal distribution was observed using the Shapiro–Wilk test. Continuous variables are expressed as mean and standard deviation or as median and interquartile range (IQR: 25th–75th percentile), depending on the results of the Shapiro–Wilk test; categorical variables are expressed as frequencies and percentages. Differences in demographic characteristics between the 117 patients included in the analysis and the 28 patients excluded from analysis were determined using the Student’s t-test or Mann–Whitney U test and the χ^2^ test. We used a generalized linear mixed model with random intercepts to analyze the change in the EQ-5D-5L over time. Patients were classified into two groups: (i) patients whose EQ-5D-5L index scores had decreased after discharge (6 or 12 months) were in the “decline group,” and (ii) patients whose EQ-5D-5L index scores had been maintained or improved after discharge (6 or 12 months) were in the “improvement/maintenance group.” Regarding patients that were available for follow-up at both 6 and 12 months, the score at 12 months was considered for the analysis. Between-group comparisons of continuous variables were performed using the Student’s t-test or Mann–Whitney U test, and the χ^2^ test was used to compare categorical variables between groups. Unadjusted and adjusted logistic regression analyses were used to determine the independent factors during hospitalization associated with a decline in QOL after hospital discharge. Variables with a *P* value < 0.05 in the unadjusted analysis and those determined to be clinically important were included in the adjusted analysis. To avoid over-fitting in the logistic regression analysis, the following three factors—EuroSCORE II [9, 16], MV for > 48 h [17], and RRT for AKI [17], —which have been shown to be associated with QOL in previous studies, were not entered into the regression model simultaneously, but each factor was separately adjusted. We used a conventional *P* value of < 0.05 to determine the level of statistical significance. Analyses were performed using IBM SPSS Statistics (version 29.0; IBM Corp., Armonk, NY, USA). In addition, a logistic regression analysis was performed with data from 145 patients with multiple imputations to account for the effect of cases excluded from the analysis (*n* = 28) because of missing data. The model assumed that data were missing at random, and included the characteristics of hospitalization and QOL after discharge. The 50 pooled imputed datasets were analyzed, and the pooled analysis results were provided by R version 4.3.2 (R Foundation for Statistical Computing).

## Results

The basic attributes of the 117 study participants included in the analysis, as well as the 28 patients excluded from the analysis because of missing data or loss to follow-up, are delineated in Supplementary Table S1. These groups differed significantly in terms of the prevalence of prior cardiovascular surgery, BI before admission, and the number of days from surgery to initial mobilization. The 117 analyzed participants, who were available for follow-up at either 6 or 12 months after discharge, had a median age of 72 years, with 69% of them being male; the median EuroSCORE II score was 3.8. The mean EQ-5D-5L index score at the time of transfer to the general ward was 0.50, the median EQ-5D-5L at discharge was 0.78. 18% of the patients in the analysis were transferred from our hospital to a rehabilitation hospital, but all had been discharged at the time of post-discharge follow-up.

**Fig. 2:**
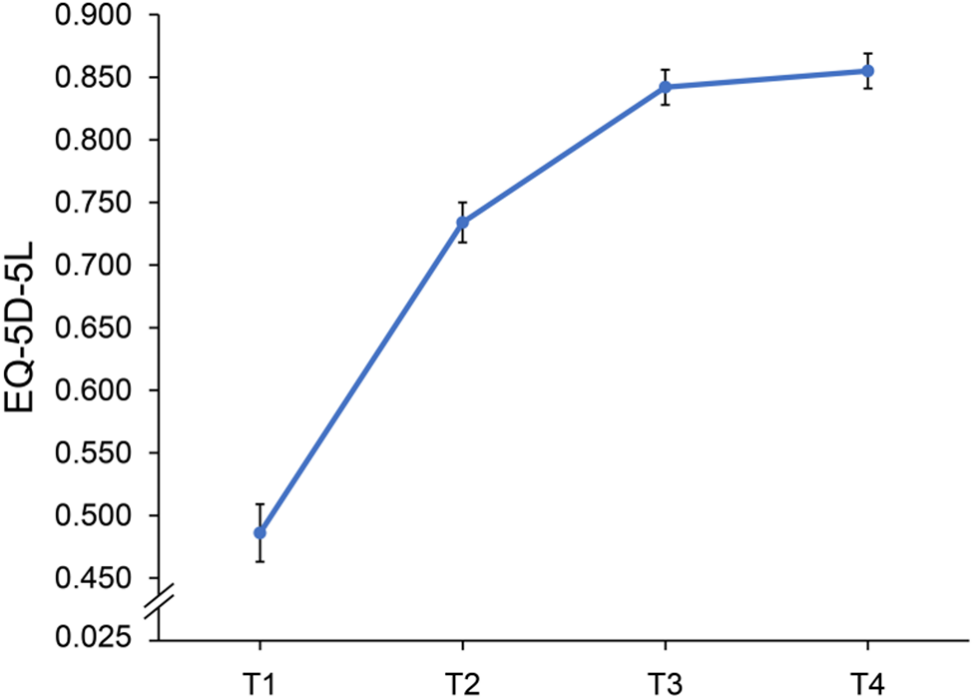
Changes in EQ-5D-5L index scores over time, measured at four different time points. The EQ-5D-5L index score ranged from 0.025 to 1.000, with higher scores indicating better health status. There was a significant improvement in scores between all time points, except between T3 and T4. Points on the line graph indicate the mean and the I-bars indicate the standard error. T1, at the time of transfer to the general ward; T2, at hospital discharge; T3, at 6 months after discharge; T4, at 12 months after discharge; EQ-5D-5L, EuroQol-5Dimension-5Level

**Fig. 3:**
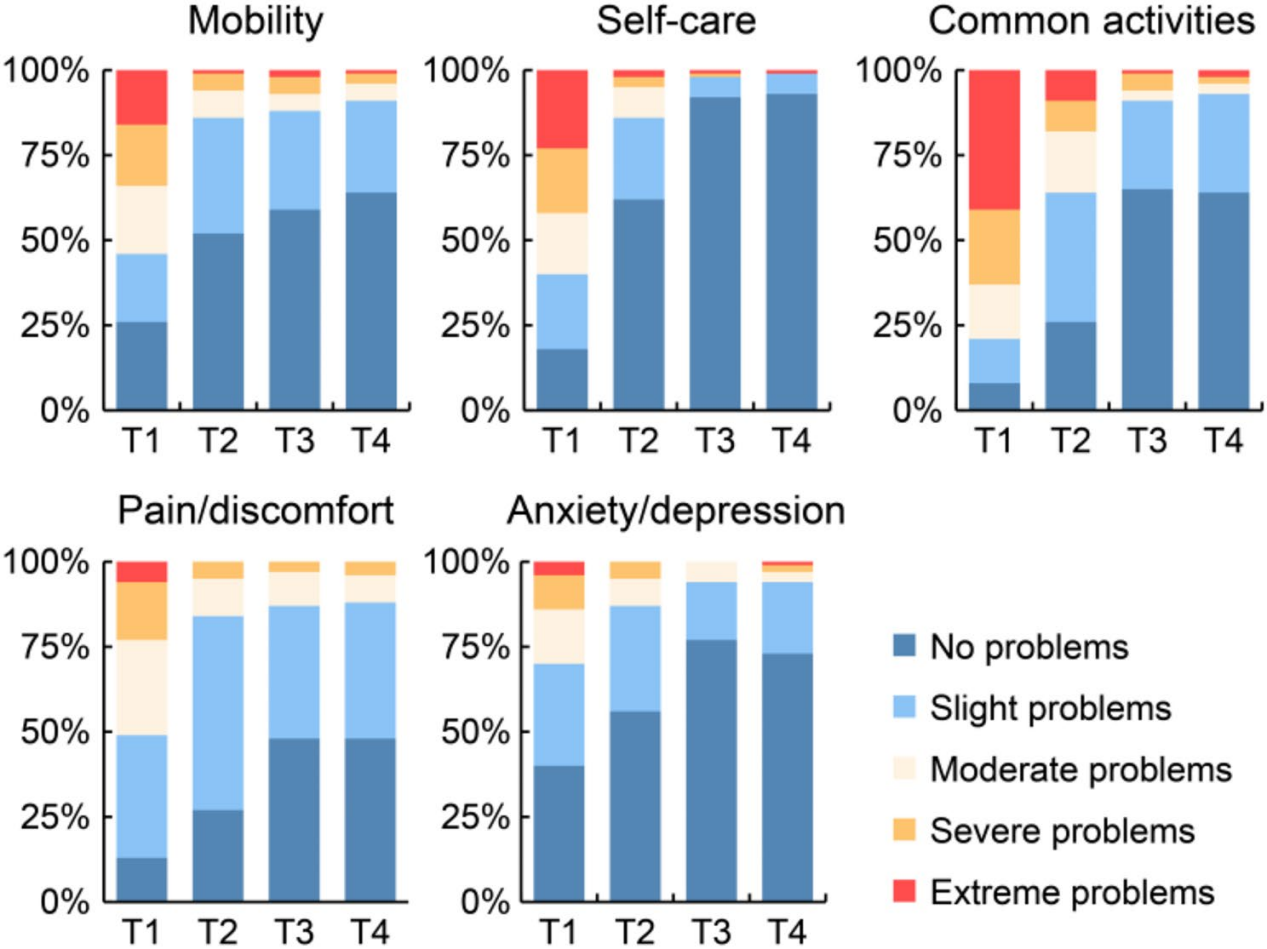
Changes over time in percentage scores for each sub-item of the EQ-5D-5L. T1, at the time of transfer to the general ward; T2, at hospital discharge; T3, at 6 months after discharge; T4, at 12 months after discharge; EQ-5D-5L, EuroQol-5Dimension-5Level

**Fig. 4:**
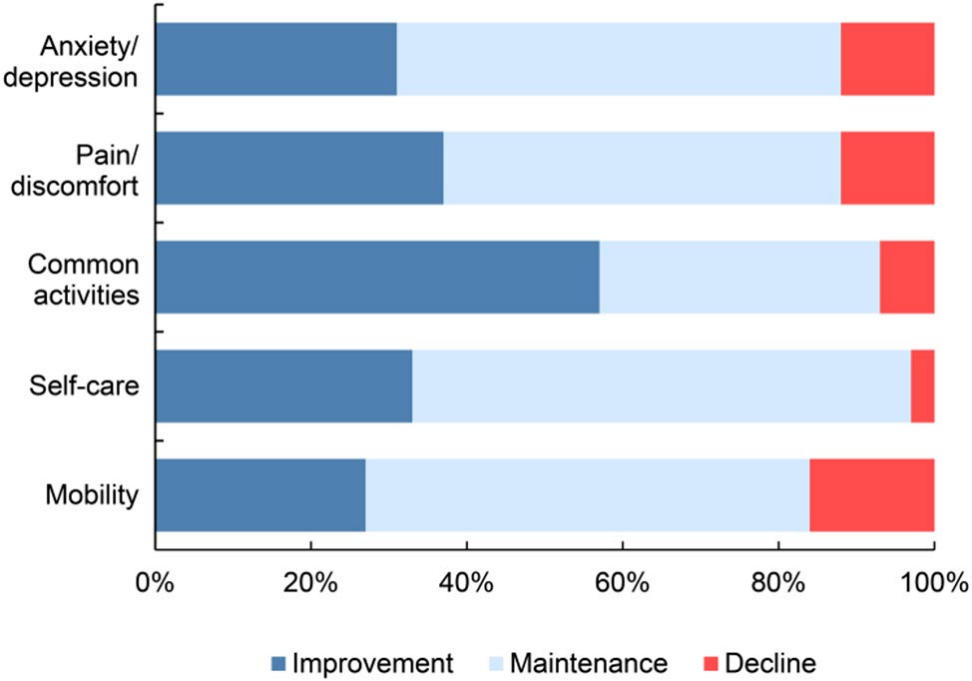
Comparison of improvement, maintenance, or decline ratios for each item of EQ-5D-5L at discharge and post-discharge. EQ-5D-5L, EuroQol-5Dimension-5Level

Figure 2 shows the temporal changes in the EQ-5D-5L index scores at four distinct time points. The EQ-5D-5L index score significantly increased over time at all time points, with the exception of the 6–12 months post-discharge period. Changes over time in the EQ-5D-5L sub-item scores are shown in Fig. 3. In all dimensions, the percentage of patients reporting “no problems” increased from the time of transfer to the general ward to 6 months post-discharge; however, there was no noticeable increase between 6 and 12 months post-discharge, while there was a slight decrease in the proportion of patients reporting “no problems” in “common activities” and “Anxiety/depression” (Fig. 3). Figure 4 shows the ratio of improvement, maintenance, or decline for each item of the EQ-5D-5L when at discharge scores were compared with post-discharge scores. Among the five dimensions of EQ-5D-5L, “common activities” was the most common item that showed an improvement in score post-discharge, followed by “Pain/discomfort” and “Self-care” (Fig. 4).

Among the 117 patients, 25 (21%) had EQ-5D-5L index scores that decreased after discharge (decline group) and 92 (79%) had EQ-5D-5L scores that were maintained or improved post-discharge (maintenance/improvement group). Table 1 shows a comparison of the characteristics during the hospitalization period between these two groups. The median age and EuroSCORE II scores were significantly higher in the decline group than those in the maintenance/improvement group (*P* = 0.029, *P* = 0.038, respectively). Furthermore, BI before admission (*P* < 0.001), preoperative Hb levels (*P* = 0.003), and serum albumin levels (*P* = 0.030) were significantly lower in the decline group than that in the maintenance/improvement group. Comparison of surgical parameters between the two groups revealed that the number of other surgical procedures was significantly higher in the decline group than that in the maintenance/improvement group (*P* = 0.031). Regarding postoperative courses, MMSE-J score before discharge was significantly lower in the decline group than that in the maintenance/improvement group (*P* = 0.032).

**Table 1:** Comparison of patient demographics during hospitalization between the two study groups based on changes in EQ-5D-5L index score post-discharge

Variables	Maintenance/improvement group	Declining group	*P* value
No. of patients, (%)	92 (79)	25 (21)	
**Preoperative status**			
Age, years	71 [63–76]	74 [71–83]	0.029^a^
Male, *n* (%)	66 (72)	15 (60)	0.259^b^
BMI, kg/m^2^	23.6 ± 3.5	24.8 ± 4.8	0.267^c^
Prior cardiovascular surgery, *n* (%)	29 (32)	7 (28)	0.735^b^
BI prior to admission	100 [95–100]	95 [88–100]	< 0.001^a^
Comorbidities (CCI)	1 [1–2]	1 [1–2]	0.687^a^
eGFR, mL/min/1.73 m^2^	60.0 ± 20.3	51.8 ± 23.5	0.086^c^
Hemoglobin level, g/dL	13.5 ± 2.0	12.1 ± 1.9	0.003^c^
Serum albumin level, g/dL	4.1 [3.6–4.4]	3.8 [3.3–4.2]	0.030^a^
Euro Score II	3.3 [1.8–5.8]	4.4 [3.5–7.3]	0.038^a^
**Surgical parameters**			
Emergent operation, *n* (%)	17 (18)	5 (20)	0.863^b^
Surgery type			
Coronary artery bypass, *n* (%)	14 (15)	2 (8)	0.352^b^
Valvular, *n* (%)	29 (31)	6 (24)	0.466^b^
Thoracic aorta, *n* (%)	29 (32)	7 (28)	0.735^b^
Combined, *n* (%)	18 (20)	7 (28)	0.362^b^
Others, *n* (%)	2 (2)	3 (12)	0.031^b^
Operation time, min	329 [276–439]	287 [239–346]	0.069^a^
CPB time, min	174 [109–233]	145 [120–189]	0.170^a^
Cross-clamp time, min	104 [69–136]	93 [72–121]	0.456^a^
Bleeding, mL	1505 [982–2603]	1538 [1113–2130]	0.897^a^
RBC transfusion, mL	1400 [840–2088]	1680 [1306–2004]	0.178^a^
APACHE II score at ICU admission	31 [27–34]	32 [29–36]	0.092^a^
**Postoperative course**			
Days from surgery to extubation	1 [1–2]	1 [1–2]	0.629^a^
Mechanical ventilation for > 48 h	14 (15)	2 (8)	0.352^b^
Dialysis for acute renal failure	4 (4)	2 (8)	0.479^b^
Days from surgery to initial mobilization, days	2 [1–3]	2 [1–3]	0.471^a^
Incidence of postoperative delirium at ICU, *n* (%)	23 (25)	10 (40)	0.139 ^b^
Length of ICU stay, days	2 [1–4]	2 [1–3]	0.781^a^
EQ-5D-5L index score at the time of transfer to the general ward	0.50 ± 0.25	0.46 ± 0.22	0.513^c^
Length of hospital stay, days	17 [13–22]	16 [14–29]	0.339^a^
SPPB total score	11 [8–12]	11 [7–12]	0.660^a^
MMSE-J score	29 [26–30]	28 [23–29]	0.032^a^
BI at discharge	100 [90–100]	95 [88–100]	0.163^a^
Home discharge from our hospital, *n* (%)	76 (83)	19 (76)	0.453^b^
EQ-5D-5L Score at discharge	0.76 [0.62–0.84]	0.83 [0.70–0.89]	0.060^a^

Data are expressed as mean ± standard deviation or median [interquartile range]

^a^Mann–Whitney U test

^b^Chi-square test

^c^Unpaired Student’s t-test

*BMI* body mass index, *BI* Barthel index, *CCI* Charlson comorbidity index, *eGFR* estimated glomerular filtration rate, *EuroSCORE* European system for cardiac operative risk evaluation, *CPB* cardiopulmonary bypass, *RBC* red blood cell, *APACHE II* Acute Physiologic and Chronic Health Evaluation II, *ICU* intensive care unit, *EQ-5D-5L* EuroQol-5Dimension-5Level, *SPPB* short physical performance battery, *MMSE-J* Mini Mental State Examination-Japanese

For the post-discharge follow-up, 105 and 106 patients responded at 6 months and 12 months, respectively (Fig. 1). Table 2 shows a comparison of the characteristics during the follow-up period between the decline group and maintenance/improvement group. FAI at 12 months after discharge was significantly lower in the decline group than that in the maintenance/improvement group (*P* = 0.014).

**Table 2:** Comparison of ADLs and EHFScBS post-discharge based on changes in EQ-5D-5L index score post-discharge

Variables	Maintenance/improvement group	Declining group	*P* value
**At 6 months (** ***n*** ** = 105)**			
No. of participants	83 (79)	22 (21)	
BI	100 [100–100]	100 [100–100]	0.472
FAI	27 [20–33]	22 [12–30]	0.088
EHFScBS	33 [26–38]	34 [24–42]	0.438
EQ-5D-5L index Score	0.89 [0.82–1.00]	0.78 [0.67–0.84]	< 0.001
**At 12 months (** ***n*** ** = 106)**			
No. of participants	84 (79)	22 (21)	
BI	100 [100–100]	100 [100–100]	0.237
FAI	29 [24–35]	23 [14–29]	0.014
EHFScBS	33 [25–39]	38 [30–42]	0.222
EQ-5D-5L index Score	0.89 [0.82–1.00]	0.72 [0.62–0.78]	< 0.001

The Mann–Whitney U test was used for analysis of data

Data are expressed as median [interquartile range]

*BI* Barthel index, *FAI* Frenchay activities index, *EHFScBS* European Heart Failure Self-Care Behavior Scale, *EQ-5D-5L* EuroQol-5Dimension-5Level

The unadjusted logistic regression analysis showed that age, BI before admission, preoperative Hb level, preoperative albumin level, and MMSE-J score before discharge were related to a decline in QOL after hospital discharge (Table 3). In the adjusted logistic regression analysis, BI prior to admission, preoperative Hb level, and MMSE-J score before discharge were independently associated with reduced QOL after hospital discharge after adjusting for Euro Score II, MV for > 48 h, and RRT for AKI, respectively (Table 4). Supplementary Tables S2 and S3 show the results of the logistic regression analysis with the multiple imputation method used to check for the effect of missing data on the results. Similarly, BI before admission, preoperative Hb level, and MMSE score before discharge were significantly and independently associated with post-discharge QOL decline in the adjusted model using multiple imputation analysis.

**Table 3:** Univariable logistic regression analysis: association of patient demographics during hospitalization with decline in EQ-5D-5L index score post-discharge (*n* = 117)

Variables	OR	95%CI	*P*
**Preoperative factors**			
Age, years	1.053	1.004–1.105	0.035
Male Sex	0.591	0.236–1.483	0.262
BI prior to admission	0.939	0.896–0.984	0.008
eGFR, mL/min/1.73 m^2^	0.981	0.960–1.003	0.089
Hemoglobin level, g/dL	0.700	0.548–0.894	0.004
Serum albumin level, g/dL	0.463	0.220–0.975	0.043
Euro Score II	1.075	0.969–1.193	0.171
**Perioperative and postoperative factors**			
Operative time, min	0.997	0.993–1.001	0.193
CPB time, min	0.998	0.994–1.002	0.360
APACHE II score at ICU admission	1.076	0.980–1.182	0.123
MV for > 48 h	0.484	0.103–2.289	0.360
RRT for AKI	1.913	0.330–11.101	0.470
Postoperative delirium at ICU	2.000	0.790–5.064	0.144
Length of ICU stay, days	0.934	0.761–1.146	0.515
SPPB total score	0.944	0.820–1.086	0.420
MMSE-J total score	0.858	0.760–0.969	0.013
BI at discharge	0.979	0.952–1.006	0.128
LOHS	1.025	0.982–1.071	0.259

*OR* odds ratio, *CI* confidence interval, *BI* Barthel index, *eGFR* estimated glomerular filtration rate, *EuroSCORE* European System for Cardiac Operative Risk Evaluation, *CPB* cardiopulmonary bypass, *APACHE II* Acute Physiologic and Chronic Health Evaluation II, *MV* mechanical ventilation, *RRT* renal replacement therapy, *AKI* acute kidney injury, *ICU* intensive care unit, *SPPB* short physical performance battery, *MMSE-J* Mini Mental State Examination-Japanese, *LOHS* length of hospital stay

**Table 4:** Adjusted logistic regression analysis: association of patient demographics during hospitalization with decline in EQ-5D-5L index score post-discharge

Variable	Adjusted for: EuroScore II			Adjusted for: MV > 48 h			Adjusted for: RRT for AKI		
	OR^a^	95%CI^a^	*P* ^a^	OR^b^	95%CI^b^	*P* ^b^	OR^c^	95%CI^c^	*P* ^c^
Age, years	1.048	0.997–1.102	0.065	1.050	1.001–1.102	0.044	1.053	1.003–1.106	0.036
BI prior to admission	0.942	0.897–0.989	0.017	0.939	0.896–0.985	0.009	0.937	0.894–0.982	0.007
Serum albumin level	0.508	0.229–1.127	0.096	0.440	0.207–0.934	0.033	0.470	0.222–0.994	0.048
Hemoglobin level	0.710	0.547–0.920	0.010	0.687	0.536–0.882	0.003	0.702	0.549–0.898	0.005
MMSE-J score	0.868	0.767–0.982	0.024	0.862	0.763–0.974	0.018	0.858	0.760–0.969	0.013

^a^Odds ratio, 95% confidence interval, and *p*-value are adjusted for EuroSCORE II

^b^Odds ratio, 95% confidence interval, *p*-value adjusted for MV > 48 h

^c^Odds ratio, 95% confidence interval, *p*-value adjusted for RRT for AKI

*OR* odds ratio, *CI* confidence interval, *EuroSCORE* European System for Cardiac Operative Risk Evaluation, *MV* mechanical ventilation, *RRT* renal replacement therapy, *AKI* acute kidney injury, *BI* Barthel index, *MMSE-J* Mini-Mental State Examination-Japanese, *EQ-5D-5L* EuroQol-5Dimension-5Level

## Discussion

This study aimed to describe the temporal changes in QOL from hospitalization to a year post-discharge in patients who had undergone cardiac and thoracic aortic surgery. Additionally, it sought to identify the factors influencing these changes over time. Although this study’s relatively small patient population from a single institution may introduce potential selection bias, we were still able to make several important findings. In our study, QOL, as assessed using the EQ-5D-5L in patients undergoing cardiac and thoracic aortic surgery, improved over time from the early postoperative period to 6 months or 1 year after discharge. To the best of our knowledge, this is the first study to determine the temporal changes in the patients’ EQ-5D-5L index score during comprehensive CR in the postoperative hospitalization period and at multiple time points after discharge. While most patients (79%) experienced an improvement in their QOL over time post-surgery, some (21%) reported a decline in QOL after being discharged from our hospital. Additionally, BI before admission, preoperative Hb level, and MMSE-J score before discharge were associated with a decline in QOL after hospital discharge in patients undergoing cardiac and thoracic aortic surgery. This relationship was also observed after adjusting for pre-existing associated factors (such as EuroSCORE II, MV for > 48 h, RRT for AKI), implying that preoperative ADL decline, Hb decline, and cognitive functional decline during hospitalization may provide additional clinical insights for considering comprehensive postoperative CR.

Recent studies have investigated changes in postoperative QOL over time. Curcio and colleagues [10] used the Kansas City Cardiomyopathy Questionnaire (KCCQ) to examine QOL at baseline and at 1 and 12 months postoperatively in patients after cardiac surgery. The study reported a marked improvement in QOL in the first month post-surgery, followed by an improvement in QOL over 12 months. Grand and colleagues [17] used the Medical Outcomes Study 36-Item Short Form Health Survey (SF-36) to examine health-related QOL at baseline and at 6 months, reporting a significant improvement in health-related QOL at 6 months. These studies, along with the present one, yielded similar results, although our study did not show a significant improvement between the 6-month and 12-month time points post-discharge. Therefore, we suggest that the period of significant postoperative QOL improvement extends up to six months post-discharge from the hospital. This can be explained by the fact that the median EQ-5D-5L index scores at 6 and 12 months after discharge were equal in the maintenance and improvement groups (Table 2). Although this study cannot conclusively determine the preoperative QOL due to the lack of preoperative QOL assessment, it can be inferred that the majority of patients returned to their original QOL level 6 months post-discharge from the hospital.

In this study, while the majority of patients available for follow-up demonstrated an improvement in QOL post-discharge, approximately 21% experienced a decline. Grand and colleagues [17] reported that at least 25% of patients experienced a marked decline in QOL compared to baseline at six months postoperatively. The patient populations in the both studies demonstrated similar trends. The median difference in EQ-5D-5L index score between the maintenance/improvement and decline groups at 6 and 12 months after discharge was 0.11 and 0.17, respectively (Table 2). The Japanese version of the EQ-5D-5L has been reported to have an estimated minimal important difference (MID) of 0.044 [27], and the MID of the EQ-5D-5L with and without disease or symptoms ranges between 0.05 and 0.10 [28]. Thus, we can confirm a clear difference in QOL values between the maintenance/improvement and decline groups in terms of the MID. This study is novel in that it reveals temporal changes in the scores for each subitem of the EQ-5D-5L. Among the five sub-items, “common activities” had the highest percentage of improvement post-discharge, followed by “pain/discomfort” and “self-care”. However, the post-discharge follow-up survey showed a trend toward significantly lower I-ADL scores at 12 months in the declining group compared to that in the QOL maintenance/improvement group (Table 2). These findings underscore the importance of post-discharge care and rehabilitation for patients who face difficulties in improving their I-ADL abilities.

Additionally, this study identified the characteristics of patients whose QOL declined after discharge from our hospital. Among the preoperative patient factors, preadmission BI and preoperative serum Hb levels were associated with a decline in QOL post-discharge after adjustment for each known factor. Hiriscau and colleagues [29] reported that cardiovascular disease patients with frailty had a significantly higher rate of ADL impairment. Zhang and colleagues [30] reported that, while anemia is a cause of frailty, frailty also contributes to anemia. Age and serum albumin levels, which reflect nutritional status, were also significantly associated in the unadjusted analysis. These factors reflect the elements of the patient’s original preoperative frailty. An association between frailty and QOL in patients with cardiovascular disease has been reported [31, 32], and a preoperative frailty component may also be associated with a decline in QOL post-discharge in patients after cardiac and thoracic aortic surgery. While previous studies have primarily examined the association between patients’ preoperative and perioperative factors and postoperative QOL, this study confirmed the association between patients’ postoperative cognitive function and changes in QOL after adjusting for known factors. Phillips and colleagues [33] reported that postoperative cognitive decline inhibited the improvement in QOL 1 year after surgery. Hogue and colleagues [34] also reported that postoperative cognitive decline is associated with a decline in QOL and I-ADL postoperatively. The post-discharge follow-up survey showed a trend toward significantly lower I-ADL scores at 12 months in the declining group compared to that in the QOL maintenance/improvement group (Table 2). While our study was not specifically designed to elucidate the underlying mechanism of this association, the link between cognitive function and I-ADL, as well as the similarities between I-ADL and QOL, could potentially have a significant impact. In a previous study by Kito and colleagues [35] on hospitalized elderly patients with heart failure, patients with I-ADL decline tended to have significantly lower MMSE scores than those who maintained them. Rodakowski and colleagues [36] reported that individuals with mild cognitive impairment have a more pronounced decline in I-ADL than patients with normal cognitive function. Previous studies [37] on community residents overseas reported an association between I-ADL and QOL. Thus, postoperative cognitive decline may have influenced the decline in the I-ADL and QOL scores after discharge. The EQ-5D-5L, which was employed as the outcome measure in this study, has been widely used in both domestic [38] and international [39] populations of patients with cognitive impairment and dementia.

Based on the results of this study, there is a clear necessity to provide ongoing comprehensive CR to patients whose QOL may decline after hospital discharge. For patients exhibiting preoperative indications of frailty, interfacility collaboration with rehabilitation hospitals in the early postoperative period [40] and transition to outpatient CR post-discharge from the hospital [41] can be effective. Furthermore, interventions targeting postoperative cognitive function [42], which were associated with QOL decline post-discharge in this study, may also contribute to improving patient outcomes.

### Study limitations

First, due to the single-center, prospective nature of this study, the sample size was relatively small in terms of the number of events of QOL decline post-hospital discharge. To circumvent overfitting, we performed a logistic regression analysis, adjusting individually for confounding variables reported in previous studies. Second, potential selection bias may have been introduced because many patients were excluded owing to failure to obtain consent, unsuitability for clinical evaluation, and missing data. To identify potential selection bias, we compared 117 patients included in the analysis (of the 145 patients from whom written consent was obtained) with the 28 patients who were excluded from the analysis due to missing data or dropout at follow-up (Supplementary Table S1). We also performed a logistic regression analysis using multiple imputation methods and confirmed that similar results were obtained (Supplementary Tables S2 and S3). Third, the patients’ preoperative QOL and mental and cognitive functions were not assessed. Although patients diagnosed with dementia or depression and bedridden patients were excluded from this study, preoperative QOL, mental health, and cognitive function may have influenced the results. Finally, we were unable to interview patients about their use of care and rehabilitation services at home post-hospital discharge. The possibility of the use of these post-discharge services and living environments may have influenced the outcome; this should be fully considered. In the future, it will be necessary to conduct a multicenter study with a larger population and an analysis that includes preoperative physical and mental functions, QOL, and post-discharge service utilization.

## Conclusion

In patients undergoing cardiac and thoracic aortic surgery, postoperative QOL demonstrated significant improvements over time, with the exception of the period between 6 and 12 months post-discharge. While the majority of patients sustained an improvement in QOL post-discharge, approximately 21% experienced a decline. Preoperative frailty-related indicators, such as ADL decline, anemia, and postoperative cognitive function, can serve as predictors of decreased QOL post-discharge. The results of the present study may serve as a useful indicator for identifying patients who would benefit from continuing comprehensive CR from phase I to phase III and for considering the content of such interventions. Given the potential selection bias introduced by the relatively small sample size in this study, future research involving larger populations is necessary.

## Electronic supplementary material

Below is the link to the electronic supplementary material.

Supplementary Material 1

Supplementary Material 2

Supplementary Material 3

## Data Availability

The datasets generated during and/or analyzed during the current study are not publicly available in order to protect personal information but are available from the corresponding author upon reasonable request.

## Abbreviations

ADL: Activities of daily living
AKI: Acute kidney injury
APACHE: Acute Physiologic and Chronic Health Evaluation
BI: Barthel Index
BMI: Body mass index
CCI: Charlson Comorbidity Index
CPB: Cardiopulmonary bypass
CR: Cardiac rehabilitation
eGFR: Estimated glomerular filtration rate
EHFScBS: European Heart Failure Self-care Behavior Scale
EQ-5D-5L: EuroQol-5Dimension-5Level
EuroSCORE: European System for Cardiac Operative Risk Evaluation
FAI: Frenchay Activity Index
Hb: Blood hemoglobin
I-ADL: Instrumental activities of daily living
ICDSC: Intensive Care Delirium Screening Checklist
ICU: Intensive care unit
IQR: Interquartile range
KCCQ: Kansas City Cardiomyopathy Questionnaire
MID: Minimal important difference
MMSE-J: Mini-Mental State Examination-Japanese
MV: Mechanical ventilation
QOL: Quality of life
RBCs: Red blood cells
RRT: Renal replacement therapy
SF-36: Medical Outcomes Study 36-Item Short Form Health Survey
SPPB: Short physical performance battery
STROBE: Strengthening the Reporting of Observational Studies in Epidemiology
