# Supplementary material for: Characteristics of longitudinal changes in quality of life and associated factors in patients post cardiac and thoracic aortic surgery: insights from a prospective cohort study
Source: J Patient Rep Outcomes. 2024 Sep 26;8:111. doi: 10.1186/s41687-024-00787-9 (PMC11427642; doi:10.1186/s41687-024-00787-9)
Supplement: Supplementary file 1 — Supplementary Material 1 [file 41687_2024_787_MOESM1_ESM.docx]

**Supplementary Files**

**Supplementary Table S1. Summary of demographic characteristics for study participants and individuals excluded from analysis because of missing data or loss to follow-up**

| **Variables** | **Missing Data** | **Consented and enrolled*** | **Study participants** | **Individuals excluded from analysis** | ***P* value** |
| --- | --- | --- | --- | --- | --- |
|  | **No.** | **(N=145)** | **(n=117)** | **(n=28)** |  |
| ***Preoperative status*** |  |  |  |  |  |
| Age, years | 0 | 71 [63–76] | 72 [65–77] | 69 [55–76] | 0.263^a)^ |
| Male, n (%) | 0 | 96 (66) | 81 (69) | 15 (54) | 0.116^b)^ |
| BMI, kg/m^2^ | 0 | 23.6 [21.2–26.6] | 23.5 [21.2–26.6] | 24.6 [20.9–26.7] | 0.800^a)^ |
| Prior cardiovascular surgery, n (%) | 0 | 39 (27) | 36 (31) | 3(11) | 0.032^b)^ |
| BI prior to admission | 0 | 100[95–100] | 100[95–100] | 100[100–100] | 0.016^a)^ |
| Comorbidities (CCI) | 0 | 1 [1–2] | 1 [1–2] | 1 [1–3] | 0.797^a)^ |
| eGFR, mL/min/1.73 m^2^ | 0 | 58.0 [47.0–72.0] | 58.0 [45.0–70.0] | 60.0 [48.8–72.0] | 0.335^a)^ |
| Hemoglobin level, g/dL | 0 | 13.2 [11.8–14.7] | 13.1 [11.8–14.6] | 13.5 [11.5–14.9] | 0.520^a)^ |
| Serum albumin level, g/dL | 0 | 4.0[3.6 - 4.3] | 4.0[3.5 - 4.3] | 4.0[3.6 - 4.3] | 0.810^a)^ |
| Euro Score Ⅱ | 0 | 3.7 [1.9–5.8] | 3.8 [1.9–6.2] | 3.1 [1.9–4.9] | 0.553^a)^ |
| ***Surgical parameters*** |  |  |  |  |  |
| Emergent operation, n (%) | 0 | 28 (19) | 22 (19) | 6(21) | 0.752^b)^ |
| Surgery type | 0 |  |  |  |  |
| Coronary artery bypass, n (%) |  | 19 (13) | 16 (14) | 3(11) | 0.677^b)^ |
| Valvular, n (%) |  | 40 (28) | 35 (30) | 5(18) | 0.200^b)^ |
| Thoracic aorta, n (%) |  | 48 (33) | 36 (31) | 12(43) | 0.222^b)^ |
| Combined, n (%) |  | 33 (23) | 25 (21) | 8(29) | 0.414^b)^ |
| Others, n (%) |  | 5 (3) | 5 (5) | 0(0) | 0.266^b)^ |
| Operation time, min | 0 | 324 [273–437] | 319 [271–437] | 334 [273–443] | 0.578^a)^ |
| CPB time, min | 0 | 167 [121–229] | 163 [114–226] | 176 [149–247] | 0.415^a)^ |
| Cross-clamp time, min | 0 | 102 [73–131] | 101 [71–133] | 102 [83–124] | 0.930^a)^ |
| Bleeding, mL | 0 | 1536[1000-2475] | 1536[1025-2383] | 1614[915-2853] | 0.802^a)^ |
| RBC transfusion, mL | 0 | 1400 [840–2117] | 1400 [840–2004] | 1630 [630–2758] | 0.581^a)^ |
| APACHE II score at ICU admission | 0 | 31 [28–34] | 31 [28–34] | 31 [26–34] | 0.334^a)^ |
| ***Postoperative course*** |  |  |  |  |  |
| Days from surgery to extubation | 0 | 1 [1–2] | 1 [1–2] | 1 [1–2] | 0.726^a)^ |
| Dialysis for acute renal failure | 0 | 6 (4) | 6 (5) | 0 (0) | 0.221^b)^ |
| Days from surgery to initial mobilization, days | 0 | 2 [1–3] | 2 [1–3] | 3 [2–3] | 0.031^a)^ |
| Incidence of postoperative delirium at ICU, n (%) | 0 | 42 (29) | 33 (28) | 9 (32) | 0.680^b)^ |
| Length of ICU stay, days | 0 | 2 [1–4] | 2 [1–4] | 3 [1–5] | 0.432^a)^ |
| EQ-5D-5L index score at the time of transfer to the general ward | 15 | 0.48 ± 0.24 | 0.50 ± 0.24 | 0.46 ± 0.22 | 0.514^c)^ |
| Length of hospital stay, days | 0 | 16 [13–23] | 17 [13–23] | 16 [12–22] | 0.273^a)^ |
| SPPB total score | 4 | 11 [8–12] | 11 [8–12] | 11 [7–12] | 0.765^a)^ |
| MMSE-J score | 8 | 28 [26–30] | 28 [25–30] | 28 [26–30] | 0.906^a)^ |
| BI at discharge | 0 | 100 [90–100] | 100 [90–100] | 95 [85–100] | 0.308^a)^ |
| Home discharge from our hospital, n (%) | 0 | 118 (81) | 95 (82) | 23 (82) | 0.908^b)^ |
| EQ-5D-5L Score at discharge | 17 | 0.78 [0.62–0.85] | 0.78 [0.62–0.85] | 0.71 [0.46–0.82] | 0.193^a)^ |

*This cohort did not include participants who died within one year of discharge (n=4).

Data are expressed as mean ± standard deviation or median [interquartile range].

a. Mann–Whitney U test

b. Chi-square test

c. Unpaired Student’s t-test

BI, Barthel Index; BMI, body mass index; CCI, Charlson comorbidity index; CPB, cardiopulmonary bypass; eGFR, estimated glomerular filtration rate; EuroSCORE, European System for Cardiac Operative Risk Evaluation; RBC, red blood cell; APACHE II, Acute Physiologic and Chronic Health Evaluation II; ICU, intensive care unit; EQ-5D-5L, EuroQol-5Dimension-5Level; SPPB, short physical performance battery; MMSE-J, Mini Mental State Examination-Japanese
