# Supplementary material for: Characteristics of longitudinal changes in quality of life and associated factors in patients post cardiac and thoracic aortic surgery: insights from a prospective cohort study
Source: J Patient Rep Outcomes. 2024 Sep 26;8:111. doi: 10.1186/s41687-024-00787-9 (PMC11427642; doi:10.1186/s41687-024-00787-9)
Supplement: Supplementary file 2 — Supplementary Material 2 [file 41687_2024_787_MOESM2_ESM.docx]

**Supplementary Table S2.** Univariate logistic regression analysis by multiple imputation: association between patient demographics during hospitalization with a decline in EQ-5D-5L index score post-discharge (n=145)

| **Variables** | **OR** | **95%CI** | ***P*** |
| --- | --- | --- | --- |
| *Preoperative* |  |  |  |
| Age, years | 1.055 | 1.005–1.107 | 0.033 |
| Male Sex | 0.598 | 0.217–1.653 | 0.325 |
| BI prior to admission | 0.937 | 0.890–0.985 | 0.013 |
| eGFR, mL/min/1.73 m^2^ | 0.982 | 0.960–1.004 | 0.109 |
| Hemoglobin level, g/dL | 0.708 | 0.562–0.891 | 0.004 |
| Serum albumin level, g/dL | 0.470 | 0.221–1.000 | 0.053 |
| Euro Score Ⅱ | 1.081 | 0.972–1.202 | 0.154 |
| *Perioperative and postoperative* |  |  |  |
| Operative time, min | 0.998 | 0.994–1.002 | 0.254 |
| CPB time, min | 0.998 | 0.994–1.002 | 0.334 |
| APACHE II score at ICU admission | 1.072 | 0.975–1.179 | 0.152 |
| MV > 48h | 0.534 | 0.106–2.698 | 0.450 |
| RRT for AKI | 1.898 | 0.314–11.459 | 0.486 |
| Postoperative delirium at ICU | 1.912 | 0.664–5.512 | 0.234 |
| Length of ICU stay, days | 0.917 | 0.741–1.133 | 0.423 |
| SPPB total score | 0.938 | 0.812–1.083 | 0.386 |
| MMSE-J total score | 0.849 | 0.749–0.963 | 0.012 |
| BI at discharge | 0.983 | 0.956–1.010 | 0.222 |
| LOHS | 1.027 | 0.983–1.073 | 0.227 |

OR, odds ratio; CI, confidence interval; BI, Barthel Index; eGFR, estimated glomerular filtration rate; EuroSCORE, European System for Cardiac Operative Risk Evaluation; CPB, cardiopulmonary bypass; APACHE II, Acute Physiologic and Chronic Health Evaluation II; MV, mechanical ventilation; RRT, renal replacement therapy; AKI, acute kidney injury; ICU, intensive care unit; SPPB, short physical performance battery; MMSE-J, Mini Mental State Examination-Japanese; LOHS, length of hospital stay
