# Supplementary material for: Characteristics of longitudinal changes in quality of life and associated factors in patients post cardiac and thoracic aortic surgery: insights from a prospective cohort study
Source: J Patient Rep Outcomes. 2024 Sep 26;8:111. doi: 10.1186/s41687-024-00787-9 (PMC11427642; doi:10.1186/s41687-024-00787-9)
Supplement: Supplementary file 3 — Supplementary Material 3 [file 41687_2024_787_MOESM3_ESM.docx]

**Supplementary Table S3. Adjusted logistic regression analysis by multiple imputation: association between patient demographics during hospitalization with decline in EQ-5D-5L index score post-discharge (n=145)**

|  | **Adjusted for : EuroScoreⅡ** | | |  | **Adjusted for : MV>48h** | | |  | **Adjusted for : RRT for AKI** | | |
| --- | --- | --- | --- | --- | --- | --- | --- | --- | --- | --- | --- |
| **Variable** | **OR^a^** | **95%CI^a^** | ***P* ^a^** |  | **OR^b^** | **95%CI^b^** | ***P^b^*** |  | **OR^c^** | **95%CI^c^** | ***P* ^c^** |
| Age, years | 1.048 | 0.997–1.102 | 0.066 |  | 1.052 | 1.003–1.104 | 0.040 |  | 1.051 | 1.000–1.105 | 0.053 |
| BI prior to admission | 0.940 | 0.893–0.989 | 0.019 |  | 0.942 | 0.896–0.991 | 0.022 |  | 0.935 | 0.889–0.984 | 0.011 |
| Serum albumin level | 0.534 | 0.244–1.171 | 0.120 |  | 0.436 | 0.207–0.919 | 0.031 |  | 0.475 | 0.224–1.006 | 0.054 |
| Hemoglobin level | 0.710 | 0.550–0.918 | 0.010 |  | 0.701 | 0.551–0.893 | 0.005 |  | 0.727 | 0.574–0.922 | 0.010 |
| MMSE-J score | 0.868 | 0.769–0.981 | 0.025 |  | 0.857 | 0.755–0.973 | 0.019 |  | 0.843 | 0.745–0.955 | 0.008 |

a: Odds ratio, 95% confidence interval, and p-value are adjusted for EuroSCORE II

b: Odds ratio, 95% confidence interval, p-value adjusted for MV>48h

c: Odds ratio, 95% confidence interval, p-value adjusted for RRT for AKI

OR, odds ratio; CI, confidence interval; EuroSCORE, European System for Cardiac Operative Risk Evaluation; MV, mechanical ventilation; RRT, renal replacement therapy; AKI, acute kidney injury; BI, Barthel Index; MMSE-J, Mini-Mental State Examination-Japanese, EQ-5D-5L, EuroQol-5Dimension-5Level
